# Dilatory effects and safety of a polyphenol-rich extract of *Steganotaenia araliacea* Hochst (Apiaceae) on rat aortic rings

**DOI:** 10.3389/fphar.2025.1642844

**Published:** 2025-09-02

**Authors:** Newton Simfukwe, Lavina Prashar, James Nyirenda, Fastone Mathew Goma, Christian Chinyere Ezeala

**Affiliations:** ^1^ Department of Physiological Sciences, School of Medicine, University of Zambia, Lusaka, Zambia; ^2^ Department of Chemistry, School of Natural Sciences, University of Zambia, Lusaka, Zambia

**Keywords:** *Steganotaenia araliacea* polyphenol-rich extract, vasodilation, isometric tension change, endothelium-dependent/and or independent vasodilation/relaxation, rat aorta rings, safety, acute toxicity

## Abstract

**Background:**

Hypertension is a major global public health concern. Many communities in developing countries still rely on medicinal plants as a source of primary healthcare for treatment of hypertension instead of conventional antihypertensive drugs. *Steganotaenia araliacea* is traditionally used to treat hypertension, but no scientific study has been conducted to prove its efficacy; hence, we carried out this work.

**Aims of the study:**

This study evaluated the vasorelaxant effect of a phenolic-rich extract from the roots of *S. araliacea* on isolated rat aortic rings.

**Methods:**

Fresh roots of *S. araliacea* collected from Chongwe district, Zambia, were dried and ground to fine powder, and polyphenols were extracted by maceration. An *ex vivo* experiment was carried out to test the *S. araliacea* polyphenol-rich extract (SAPE) on isolated rat aortic rings. Isometric tension measurements on the aortic rings were evaluated to study the vasodilatory effects using the tissue/organ bath and the PowerLab data acquisition system. Phenylephrine (PE) was used for pre-contraction of the aortic rings, and cumulative concentrations (0.2 mg/mL to 16.91 mg/mL) of SAPE were tested to antagonize the aortic contraction. Acetylcholine (ACh) and sodium nitroprusside (SNP) served as the standard drugs for inducing dilatory effects against PE. L-nitro-arginine-methyl-ester (L-NAME) was applied to block endothelial nitric oxide synthase (eNOS) activity for establishing the possible involvement of the NO/cGMP mechanism of action.

**Results:**

The total polyphenol content ranged from 952 ± 3.40 mg gallic acid equivalent (GAE/100 g) to 97.8 ± 1.20 mg GAE/100 g of dry extract. The methanolic extract induced significant dilatory effects on the endothelium intact and denuded aortic rings, with the maximum percentage relaxation of 98.9% ± 0.714% and 97.29% ± 3.34%, respectively. The median IC_50_ values were 5.07 ± 1.05 mg/mL and 5.56 ± 1.08 mg/mL, respectively. Vasodilatory effects were significantly reduced in the presence of L-NAME, with p value < 0.05. The aqueous root extract was found to be practically nontoxic at a concentration of 10,000 mg/kg in mice.

**Conclusion:**

This study demonstrated that the polyphenol-rich extract of *S. araliacea* produces significant vasodilatory effects, demonstrating potential to act as an antihypertensive agent. Further studies are needed to validate these findings.

## Graphical Abstract

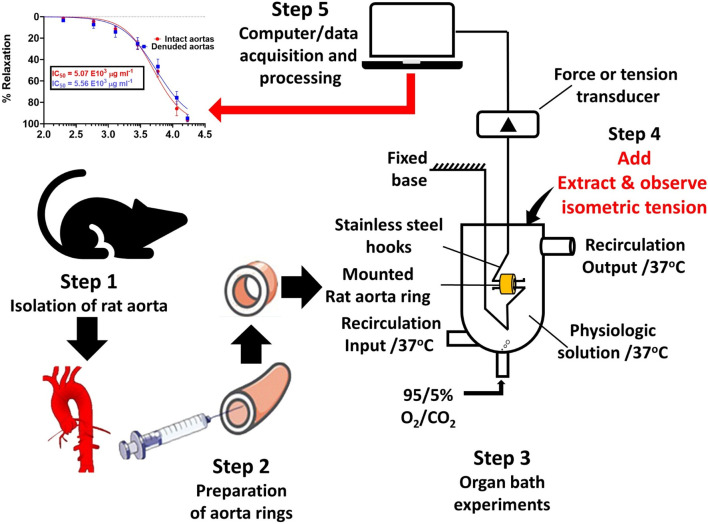

## 1 Introduction

Hypertension is a major public health concern, and the burden of untreated and uncontrolled conditions remains high because of the complex pathophysiology of this disorder. In Zambia, the prevalence of hypertension among adults aged ≥ 18 years is 30.7% (Fastone et al., 2021). In urban Lusaka, the prevalence rate is 34.8% (Goma et al., 2017). A cross-sectional study conducted by Kathy Lynn et al. (2018) revealed the prevalence rate of 46.9% among rural Zambians of the western province.

Despite the development and approval of conventional antihypertensive drugs for managing this disorder, many communities in Africa still rely on traditional medicines for treatment (Lassale et al., 2022; Nyirenda and Chipuwa, 2024). For example, metabolites derived from some herbs and spices such as *Panax ginseng* have been proven to reduce hypertension by exerting antioxidant properties and generating nitric oxide (NO) via the stimulation of the eNOS-NO signaling pathway (Liu and Huang, 2016). *Steganotaenia araliacea* is used in folk medicine, and it has been extensively studied for its medicinal properties. It is found in many tropical regions of Africa and is generally referred to as carrot tree. Other plants such as *Anacardium occidentale* (Nyirenda and Chipuwa, 2024; Nyirenda et al., 2024) have also been shown to have a variety of uses, including the treatment of diabetes and hypertension.

In Zambia, the plant is referred to as “*Fyopola*” by the locals of the Chongwe district and is reportedly used in folk medicine as a uterotonic agent to enhance parturition during labor (Hajj Magalie et al., 2020) and for treating various other conditions (Pharaoh et al., 2021) including hypertensive disorders and other cardiovascular diseases (CVDs) in humans (Andemariam, 2010).

Phytochemical investigation and structural characterization studies of *S. araliacea* extracts have revealed the presence of different classes and sub-classes of polyphenols. For example, one particular study showed the presence of two flavonoids that were structurally identified as apigenin-4-glucoside-1 and sophoraflavone B2 (Omolo et al., 2014). The CG-MS and LC-SPEC-NMR analyses of the methanol extract of *S. araliacea* seeds and stem bark showed the presence of other classes of polyphenolic compounds identified as apiol (5-Allyl-4,7-dimethoxy-benzo(1,3) dioxole), scoparone (6,7-dimethoxycourmarin), stigmasterol, and falcarinol (Mussie Sium et al., 2014; Rica et al., 2015). In Zimbabwe, other polyphenols identified as anthocyanins and tannins were found in the bark of *S. araliacea* (Mazuru, 2019).

The mentioned active polyphenolic principles, extracted and purified from several other medicinal plants, are beneficial to mammalian blood vessels. They have been shown to significantly reduce arterial stiffness and enhance the compliance and function of the vascular smooth muscle (Mona et al., 2020). For example, apigenin-4-glucoside is a phenolic metabolite that is reported to protect endothelial-dependent relaxation against oxidative stress and ameliorate endothelial dysfunction by increasing the bioavailability of NO in rat aortas (Bi-hui et al., 2009; Yong-He et al., 2000).

Vascular smooth muscle cells play an integral part in the maintenance of vascular homeostasis through active contraction and relaxation (Gao, 2022). Hypertension and associated CVDs are reported to compromise the integrity of the vascular smooth muscles by reducing the compliance of peripheral arteries as well as increasing the stiffness of blood vessel walls (Cavalcante et al., 2011). One of the major underlying cause is the reduced generation of relaxing factors such as NO and prostacyclin from the endothelial cells of blood vessels and increased oxidative stress and/or reactive oxygen species (ROS), leading to endothelial cell injury and dysfunction and resulting in various forms of hypertensive disorders (Touyz et al., 2018).

Studies have shown that interventions aimed at improving the vascular function, either with traditional or conventional remedies, can be safe and potentially effective. However, the effects of *S. araliacea* polyphenols on vascular smooth muscle remained unclear. Therefore, the present study sought to investigate the vasodilatory effects of crude total polyphenols extracted from *S. araliacea* on isolated rat aorta rings in order to substantiate the usage of *S. araliacea* in the management of hypertension.

## 2 Materials and methods

### 2.1 Chemical reagents and drugs

Phenylephrine (PE), acetylcholine chloride (ACh), sodium nitroprusside (SNP), NG-nitro-L-arginine methyl-ester (L-NAME), and gallic acid were procured from Merck (Sigma-Aldrich Co.), whereas Folin–Ciocalteu (F–C) reagent, hexane, chloroform, ethyl estate, acetone, methanol, and ethanol were purchased from HiMedia. Pentobarbital sodium was procured from Livestock Services Cooperative Society, Lusaka, Zambia.

### 2.2 Plant material collection and identification

*S. araliacea* Hochst (Apiaceae) root materials were collected from their natural habitat at the University of Zambia (UNZA), Liempe Farm (located at 15° 24ʹ south and 28° 28ʹ east at 1,171 m above sea level), in Chongwe region, Lusaka province, Zambia. Taxonomic identification of the plant was done by UNZA, School of Natural Sciences, Biological Sciences Department Herbarium, where a voucher specimen (No. 22289) was deposited.

### 2.3 Preparation of the plant material and polyphenol extraction

Approximately 1 kg of dried and finely ground root bark of *S. araliacea* was macerated three times in 20% methanol under stirring, as described by Vivian Caetano et al. (2014). Other extracting solvents included ethanol, acetone, hexane (absolute), and chloroform (absolute). The extracts were then discretely filtered, and, for phase separations, the filtrates were successfully partitioned in chloroform to separate the aqueous portions containing polyphenols from other nonpolar ones using separating funnels (Nino et al., 2016). The aqueous portions were concentrated to dryness at 40 °C using a rotary evaporator to yield the crude polyphenol-rich extracts from methanol, ethanol, and acetone.

### 2.4 Determination of the total polyphenolic content

The total polyphenolic content was estimated by the F–C spectrophotometric procedure, as described by Singleton Vernon et al. (1999) and revised by Sánchez-Rangel et al. (2013). This method is dependent on the reduction–oxidation reaction between phenolic compounds and sodium carbonate to form phenolates, which in turn reduces the yellow F–C reagent (phosphomolybdic/phosphotungstic acid) to form blue complexes. The intensity of this complex is spectrophotometrically determined to estimate the total polyphenolic content by reading the absorbance (ABS) at 760 nm.

**FIGURE 1:**
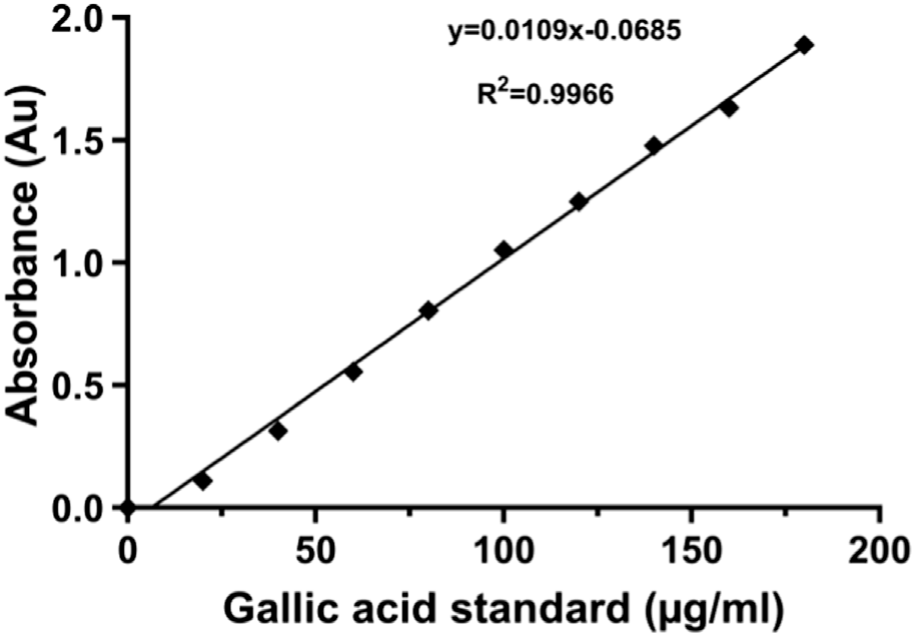
Gallic acid standard curve with Folin–Ciocalteu reagent.

Dry polyphenolic plant extracts were reconstituted in the respective extracting solvents to form solutions of known concentrations. The F–C reactions were then induced by adding 0.5 mL of the extract to 2.5 mL of 10% F–C reagent and 2.5 mL of 7.5% sodium carbonate, and the solution was incubated at 45 ^o^C for 30 min before reading the absorbance with a UV–visible spectrophotometer. The reconstituting solvents without any extracts were used as negative controls. All the samples were prepared in triplicates, and the mean values of absorbance (ABS) readings were computed for the determination of gallic acid equivalents. Total polyphenolic contents (TPCs) were then expressed as mg of gallic acid equivalent (GAE) per 100 g of dry powdered plant extract from a 10-point gallic acid standard calibration curve ranging from 0 μg/mL to 180 μg/mL (Figure 1).

The TPCs of the extracts described were calculated from the mean values of optical density (O.D.) triplicate readings by using the following formula:

$$C=C_{1}\;x\;V/m,$$

where C = TPC in mg/g GAE (gallic acid equivalent), C_1_ = concentration of gallic acid established from the standard calibration curve in µg/mL (y = 0.01x - 0.068: r^2^ = 0.996; y is the absorbance; x is the solution concentration), V = volume of extract in ml, and m = the weight of the plant extract in grams.

The TPC was expressed in mg of gallic acid equivalent (GAE)/100 g of the powdered crude plant extract, and the study narrowed down to a single extracting solvent combination that yielded the highest polyphenolic content for studying the vasorelaxant effects on isolated rat aortas.

### 2.5 Laboratory animals

Male Wistar rats weighing between 180 g and 250 g housed in the animal unit under the Physiological Sciences Department at the University of Zambia, were kept in a 12-h light/dark cycle and given free access to standard animal feed pellets with clean drinking water, as guided by the Institute for Animal Care and Use Committee published by the US national Institutes of Health (Council National Research et al., 2011). Wistar albino rats are a preferred laboratory animal model because of their anatomical, genetic, and physiological similarities to humans, and they are easy to handle and maintain (Bryda, 2013).

### 2.6 Acute toxicity test (LD_50_ determination)

Acute toxicity tests were carried out using mice. The method described by Bernas et al. (2004) was used to determine the approximate lethal dose (ALD). For this, 12 mice were randomly separated into six groups of two mice each. The crude extract was dissolved in water and administered orally to the mice using 22 G metal gavage feeding needles and syringes. Starting at 10 mg/kg, the doses were logarithmically increased at 0.6 log units, corresponding to the following doses—10, 40, 160, 630, 2,512, and 10,000 mg/kg. The animals were observed overnight, and the number of deaths in each group was recorded. The highest dose that caused no deaths and the next higher dose at which death occurred defined the lower and upper limits, respectively, of the approximate lethal dose. Using the ALD as a guide, the actual LD_50_ was determined in a set of 25 mice. These were separated into five groups of five mice each, and five doses were chosen between the upper and lower limits of the ALD as follows: 1,000, 2,000, 5,000, 7,500, and 10,000 mg/kg. These were administered orally to the five respective groups. The animals were left overnight, and after that, the number of deaths in each group was recorded, and the LD_50_ was determined by probit analysis (Akçay, 2013) using SPSS 21 (IBM Corporation, Armonk, New York).

### 2.7 Vascular reactivity setup

Male Wistar albino rats (180 g–250 g) were anesthetized with an allowable dose of 60 mg/kg of pentobarbital sodium and sacrificed by cervical decapitation in accordance with the Institutional Animal Care and Use Committee guidelines (Welfare National Institutes of Health, 2002), and a segment of the descending thoracic aortas was painstakingly excised and immediately placed in aerated Krebs–Henseleit buffer physiological salt solution (PSS) containing NaCl, 130 mM; KCl, 4.7 mM; CaCl_2_, 1.6 mM; MgSO_4_, 1.2 mM; KH_2_PO_4_, 1.2 mM; NaHCO_3_, 14.9 mM; EDTA, 0.026 mM; and glucose, 5.5 mM at pH 7.4, in accordance with the procedure (Fredalina et al., 2018).

Fat and all the connective tissues were removed, and the aortic tissues were segmented into 3–4-mm rings. Each aortic ring was suspended in a 25-mL organ bath chamber by means of two (2) L-shaped stainless steel hooks running parallel through the aortic lumen. In order to record and measure the isometric tension change, the upper hook was tied to one end of a thread, and the other end was tied to the isometric force transducer (model: MLT 210/A, AD Instruments, Australia), which, in turn, through a signal amplifier-ML301, was connected to the PowerLab 26T Data Acquisition unit (model: ML 856, AD instruments, Australia), and LabChart version 8 software was used for data recording. The temperature of Kreb’s solution in the organ bath chambers was maintained at 37 ^o^C with constant aeration. The aortic ring tissues were allowed to equilibrate for 45 min and washed three times at 15-min intervals, and thereafter, the resting baseline tension was set at 30 mN. In order to ascertain the viability of the aorta rings, the normal PSS (Krebs–Henseleit) was substituted with high potassium PSS (60 mM K^+^ PSS), and the rings were allowed to constrict to a steady “plateau” tension before washing them off with normal PSS to restore the resting baseline tension.

The denudation of the aortic endothelium for endothelial independent relaxation bioactivity was achieved by gently rubbing against the luminal surface with a cotton material tied around a thin stainless wire before suspending the ring segments into organ bath chambers. Endothelial denudation was confirmed by graded concentrations of the endothelial dependent agonist, acetylcholine, which could only produce an average vasorelaxation of less than 10% against phenylephrine (PE) precontracted aortic tissue.

### 2.8 Experimental design

#### 2.8.1 Effect of standard ACh and SNP on contractions induced by PE

**FIGURE 2:**
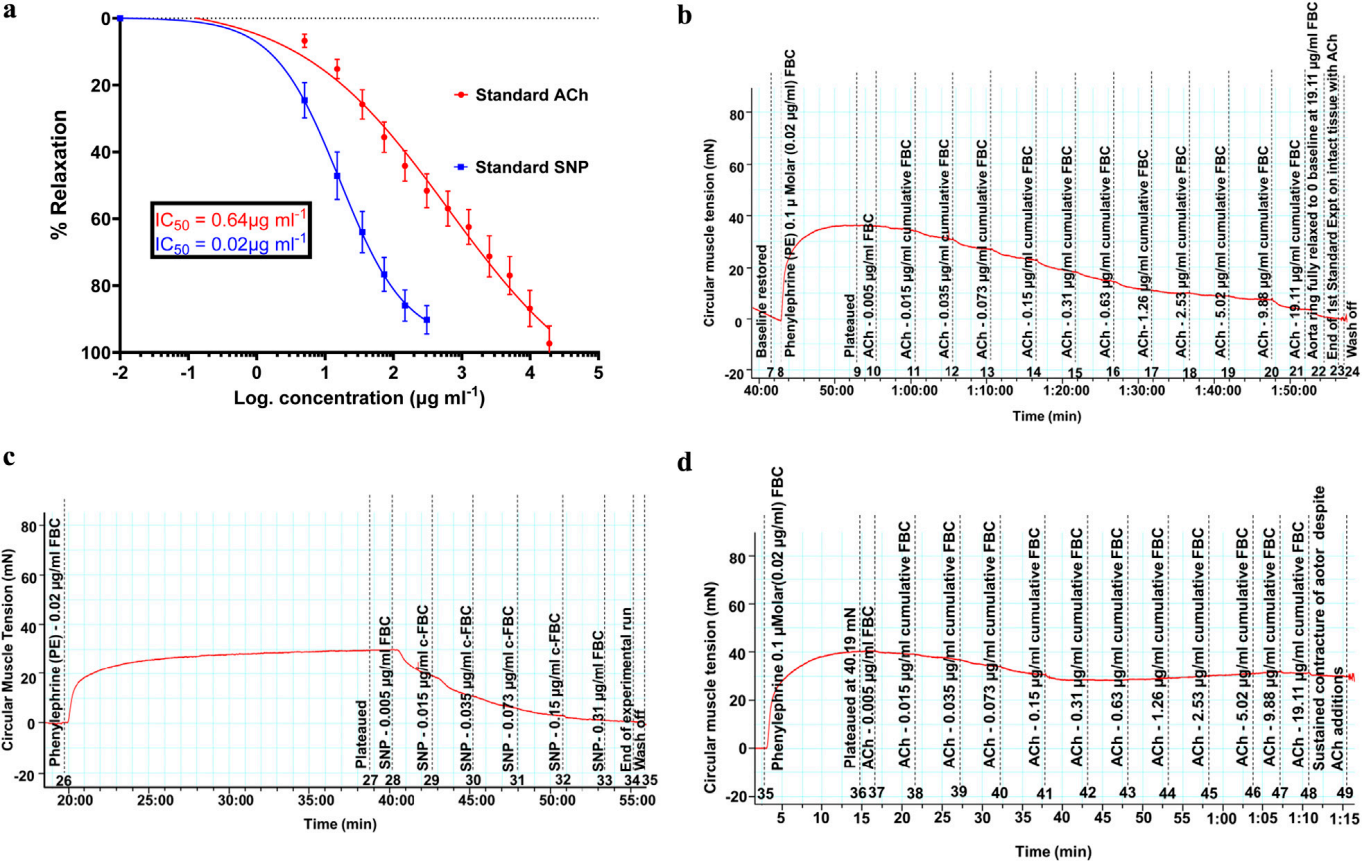
**(a)** Vasodilatory effects of standard acetylcholine and sodium nitroprusside showing the standard concentration (dose)–response relationships of vasodilation induced in endothelium-intact and denuded rat aortas, respectively. Spearman’s correlation coefficients of r = 0.997 and 0.996, respectively, show a strong association of an increase in the concentration with increasing vasodilation (vasorelaxation). P-value less than 0.0001 showed significance. Values are expressed as mean ± SEM of six experiments (n = 6 rings). **(b)** Trace of reducing isometric tension, signifying vasodilation (relaxation), because of the standard acetylcholine cumulative doses ranging from 0.005 μg/mL up to 19.11 μg/mL on the intact endothelium of rat aortas. **(c)** Trace of reducing isometric tension, signifying vasodilation (relaxation), because of the standard sodium nitroprusside cumulative doses ranging from 0.005 μg/mL up to 0.31 μg/mL on the denuded endothelium of rat aortas. **(d)** Sustained aortic tissue contracture despite the presence of acetylcholine doses. The pre-contracture lasted for over 1 h, and the recorded tension drop was less than 10%.

PE (0.02 μg/mL) (Fredalina et al., 2018), an α1-adrenergic receptor agonist, was introduced to constrict the aortas until the tension “*plateaued*,” as in the case of 60 mM K^+^PSS. Two-fold graded serial concentrations of standard acetylcholine (ACh) were then cumulatively added at 5-min time intervals to the final organ bath concentration (FBC) of 0.005 μg/mL and increased up to 19.11 μg/mL (shown in Figure 2b). The recorded isometric tension changes were computed for the construction of an endothelium-dependent dose–response relationship curve. For endothelial-independent vasorelaxation, sodium nitroprusside was applied with the serial grading concentration ranging from 0.005 μg/mL up to 0.31 μg/ml, as shown in Figure 2c. The experimental procedures on both intact and denuded aortic rings were replicated six times on different aortic rings.

#### 2.8.2 Effect of SAPE on endothelium intact and denuded aortas

The aqueous-methanolic (80:20 %v/v) dry polyphenolic extract was re-constituted in normal physiological salt solution (Krebs–Henseleit buffer) as a stock concentrate to be applied for further dilution in the 25-mL organ bath chamber. Rat aorta ring preparations were pre-contracted with 0.1 µM (0.02 μg/mL) PE, and upon reaching sustained “plateau” tension, two-fold serial graded concentrations of *S. araliacea* polyphenols were cumulatively introduced to the FBC ranging from 0.2 mg/mL to 16.91 mg/mL at 5-min intervals. The selection of the graded concentrations was based on the concentration–response relationship and receptor occupation concept (Chung-Chuan et al., 2010; Yagiela et al., 2010). Vasodilatory effects of SAPE were assessed by changes in isometric tension. The change was calculated as a percentage of sustained contraction by 0.1 µM (0.02 μg/mL) PE, and the effective concentration at 50% of the maximum dilation (relaxation, IC_50_), extrapolated from the concentration (dose)–response curve, was used for comparative analysis between endothelium-intact and denuded aortas. This was further investigated to characterize a possible mechanism of action involving the generation of NO from endothelial cells. To achieve this objective, L-nitro-arginine-methyl-ester (L-NAME) was applied to endothelial intact aortas to block the endothelial NO synthase activity from releasing NO from L-arginine. Six replicates of the experiments were performed on both the intact and denuded-endothelium aortic ring segments to study the vasodilatory effects of SAPE on vascular smooth muscles.

### 2.9 Data analysis

Values were expressed as means ± standard error of the mean (SEM). Experiments on TPC were performed in triplicates, and a standard gallic acid calibration curve was constructed to obtain linear regression equation for analysis of TPC. Relaxant responses (isometric tension change) to TPC were normalized and expressed as percentage relaxation against the PE-induced maximal contraction level (plateau tension). The reversal of PE pre-contracture by SAPE (antagonist) to zero baseline tension was considered 100% relaxation. The concentration (dose)–response curves were constructed using a non-linear curve-fit model to obtain IC_50_ values, and the relationships were analyzed using Spearman’s correlation coefficient test. Each point of the plotted graph is represented by mean ± SEM of six replicates of the experiments. Values of p < 0.05 were considered significant on the applied statistical tools (Mann–Whitney t-test for analyzing the difference in the presence and absence of L-NAME and for endothelial denuded and non-denuded aortas in response to SAPE) using GraphPad prism software version 10.3.0. The IC_50_ values were obtained by the variable sigmoid slope of the concentration–response curves.

## 3 Results

### 3.1 Analysis of the total polyphenolic content of *Steganotaenia araliacea*

The F–C method of polyphenolic content analysis (Figure 1) revealed varying concentrations of total polyphenols extracted with solvents of varying polarities, as shown in Table 1. The methanolic extract yielded the highest TPC estimated as 952.00 ± 3.4 mg GAE/100 g of dry root powdered material of *S. araliacea* plant. Chloroform contained the least polyphenol content yield of 97.78 ± 1.2 mg GAE/100 g. Therefore, the methanolic extract was preferred for subsequent investigations of studying the vasodilatory effects on rat thoracic aortas.

**TABLE 1:** Total polyphenol content yields.

Extracting solvent	Percentage yield (%)	Gallic acid (mg/mL)	TPC, mg GAE/100 g
Methanol, 20%	17.40 ± 1.12	0.714 ± 0.006	952.00 ± 3.4
Ethanol, 20%	15.75 ± 0.36	0.652 ± 0.003	869.33 ± 2.9
Acetone, 20%	19.85 ± 0.81	0.631 ± 0.007	841.28 ± 3.1
Hexane	1.10 ± 0.17	0.033 ± 0.002	103.41 ± 1.6
Chloroform	0.36 ± 0.09	0.018 ± 0.001	97.78 ± 1.2

### 3.2 Acute toxicity (LD_50_) studies

None of the mice died at any of the six doses used for the ALD or the five doses used for the actual LD_50_ determination. Therefore, the LD_50_ was estimated to be greater than 10,000 mg/kg. This value is much higher than previously reported values from similar studies that utilized the stem-bark of the plant. For example, Agunu et al. (2003) and Agunu et al. (2005) reported an oral median lethal dose value of 1,750 mg/kg for the stem-bark of *S. araliacea*. This difference may be because of the use of different plant parts (the stem-bark) in those studies or because of the environments in which the plants grew. For other plant species, LD_50_ values reported in literature vary widely (Onwusonye et al., 2014; Vaithiyanathan and Mirunalini, 2015). The researchers believe that this is the first report on the safety of the root extract of *S. araliacea*. Based on the Hodge–Steiner toxicity scale (Hodge Harold and Sterner James, 1949), substances with oral LD_50_ values between 5,000 mg/kg and 15,000 mg/kg in rats could be regarded as “Practically Non-toxic.” Based on this, it was concluded that the aqueous root extract of *S. araliacea* harvested in Zambia was practically non-toxic to rodents. The extent to which this conclusion could be extrapolated to humans is unclear.

### 3.3 Vasodilatory effects of acetylcholine and sodium nitroprusside standards

Acetylcholine was applied as a standard because it is associated with endothelial-dependent effects in terms of vascular smooth muscle relaxation, although physiologically, it is not a significant contributor to arterial tone as there is no parasympathetic supply to the arterial muscle. However, acetylcholine causes vasodilation by releasing NO from the endothelial cells. NO rapidly diffuses to adjacent smooth muscle cells, where it activates the soluble guanylate cyclase (sGC) with the generation of cyclic guanosine monophosphate (cGMP), resulting in decreased [Ca^2+^] and induction of vasodilation (Aamer et al., 2010). On the other hand, sodium nitroprusside acts directly on the vascular smooth muscle by reacting with tissue sulfhydryl groups with the release of NO (Bonaventura et al., 2008).

The vasodilatory effects of the standard ACh and SNP are presented in Figure 2a, and their attained maximum (E_max_) percentage relaxations were evaluated as 96.36% ± 0.31% and 91.41% ± 0.14% at the cumulative doses of 19.11 μg/mL and 0.31 μg/mL, respectively. The IC_50_ values for the standards were extrapolated as 0.64 μg/mL and 0.016 μg/mL. Figures 2b,c demonstrate parts of the actual traces of data recordings, showing isometric tension reduction signifying vasodilation. Figure 2d confirms the denudation of the aortic endothelium by its sustained contracture despite the additions of acetylcholine doses.

#### 3.3.1 Trace of isometric tension change due to the ACh relaxation effect

Figure 2b shows the trace of isometric tension change due to the relaxation effects of acetylcholine.

#### 3.3.2 Trace of isometric tension change due to the SNP relaxation effect

Figure 2c shows the trace of isometric tension change due to the relaxation effects of sodium nitroprusside.

#### 3.3.3 Trace of sustained isometric tension of the aortic ring contracture

Figure 2d shows the trace of sustained isometric tension of the aortic ring despite presence of acetylcholine.

### 3.4 Vasodilatory effects of SAPE

**FIGURE 3:**
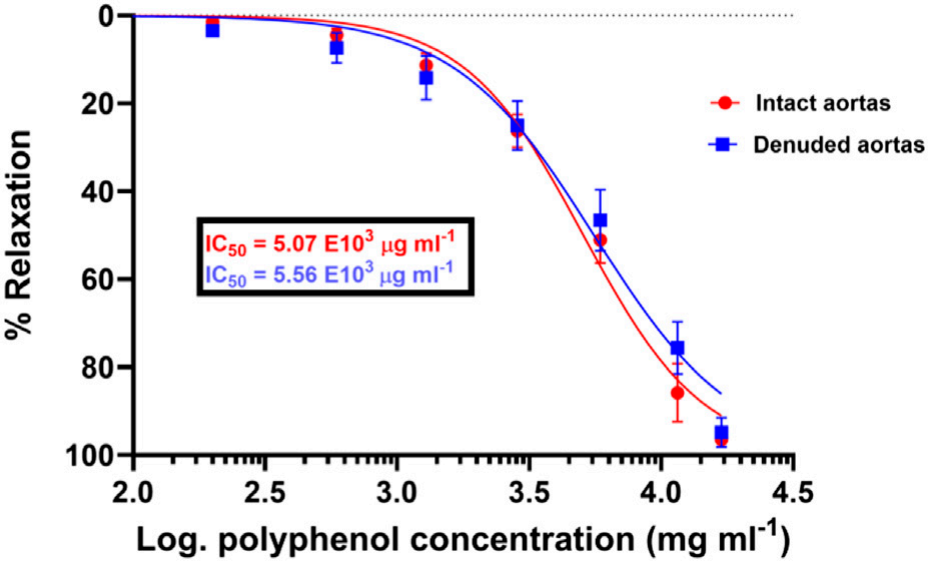
Concentration (dose)–response curves of vasodilation induced by the total polyphenolic extract of *S. araliacea* on endothelium-intact aortas with a slightly reduced pharmacological potency of SAPE on denuded rat aortas because of increased IC_50_ values. Spearman’s correlation coefficients of r = 0.996 and 0.994 show a strong association of an increase in concentration with increasing vasodilation (vasorelaxation), with p-value < 0.0001. Values are expressed as mean ± SEM of six experiments (n = 6 rings).

**FIGURE 4:**
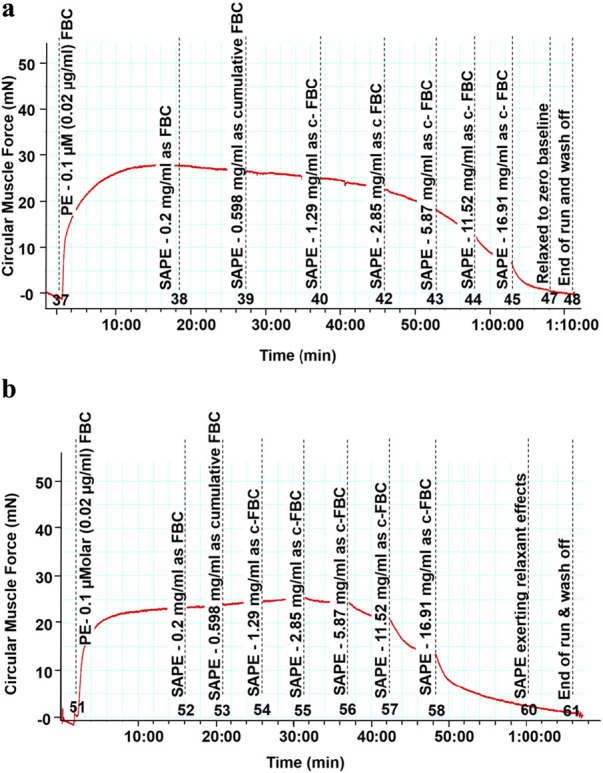
**(a)** Trace of reducing isometric tension, signifying vasodilation (relaxation), because of the cumulative doses on endothelium-intact thoracic rat aortas with the starting dose of 0.2 mg/mL up to the last dose of 16.91 mg/mL, which were added cumulatively in the organ bath. **(b)** Trace of reducing isometric tension, signifying vasodilation (relaxation), because of the cumulative doses on endothelium-denuded thoracic rat aortas with the starting dose of 0.2 mg/mL up to the last dose of 16.91 mg/mL, which were added cumulatively in the organ bath.

The methanolic polyphenol-rich extract of *S. araliacea* (0.2–16.91 mg/mL FBC: TPC = 4.72 mg GAE/g) induced concentration-dependent vasodilatory effects on both endothelium-intact and denuded rat thoracic aortas that were pre-contracted with 0.02 μg/mL of PE (0.1 µM). The maximum effects (E_max_) in terms of the vasodilation percentages were recorded as 98.90% ± 0.714% and 97.29% ± 3.34% at the total cumulative concentration of 16.91 mg/mL, respectively. The derived median IC_50_ values of the SAPE on endothelium-intact aortas was 5.07 ± 1.05 mg/mL (1.44 mg GAE/g), while that of endothelium-denuded aortic rings was recorded as 5.56 ± 1.08 mg/mL (1.58 mg GAE/g), as shown in Table 2 and Figure 3. The effective range and IC_50_ values of SAPE appear to be very high compared to those of the standard ACh or SNP. This huge discrepancy is attributed to the presence of several other compounds of the crude extract, which may not impact any bioactivity of interest. Experimental trace recordings of the isometric tension change in Figures 4a, b demonstrate the induction of vasodilations (relaxations) of the isolated rat aortas by SAPE.

**TABLE 2:** Vasodilatory effects of SAPE and standard drugs (ACh and SNP) on aortic preparations.

Polyphenolic extract/standard drugs	Endothelium-intact		Endothelium-denuded	
	E _max_ (%)	IC_50_ (µg/mL)	E _max_ (%)	IC_50_ (µg/mL)
*S. araliacea* (SAPE)	98.90 ± 0.71	5.07 × 10^3^ ± 1.05	97.3 ± 3.34	5.56 × 10^3^ ± 1.08
Acetylcholine (ACh)	96.36 ± 0.31	6.40 × 10^−1^ ± 1.16	N/A	N/A
Sodium nitroprusside (SNP)	N/A	N/A	91.4 ± 0.14	1.60 × 10^−2^ ± 1.14

#### 3.4.1 Experimental trace recording of isometric tension change

Figure 4a shows the trace of isometric tension change on endothelium intact aortic rings as a result of cumulative doses of the SAPE in the 0.2 to 16.91 mg/ml concentration range.

#### 3.4.2 Experimental trace recording of isometric tension change

Figure 4b shows the trace of isometric tension change on endothelium denuded aortic rings as a result of cumulative doses of the SAPE in the 0.2 to 16.91 mg/ml concentration range.

#### 3.4.3 Effect of L-NAME on the vasodilatory effect of SAPE

**FIGURE 5:**
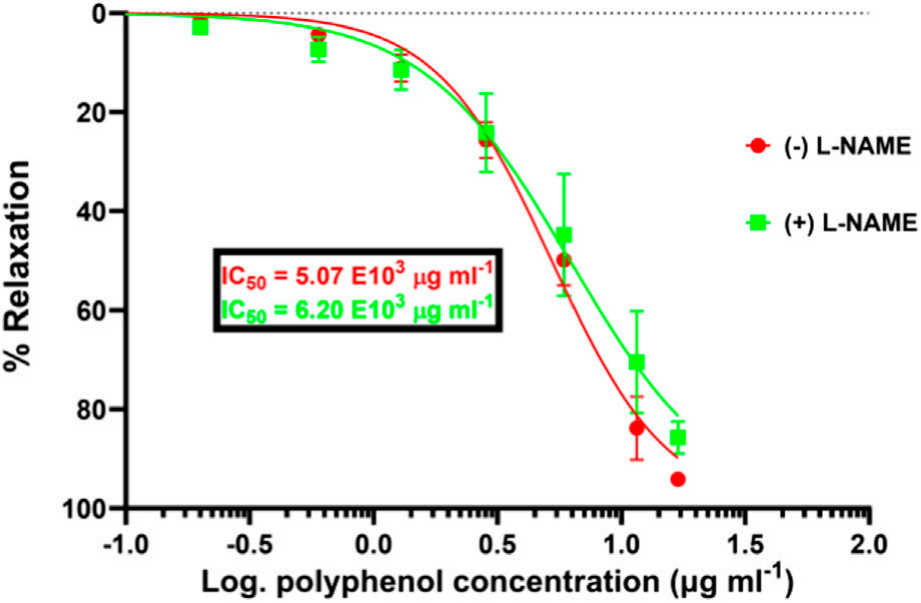
Significantly reduced pharmacological efficacy of SAPE in the presence of NO synthase inhibitor (L-NAME) compared to that in the absence of L-NAME by the increased IC_50_ value from 5.07 to 6.20 mg/mL. Mann–Whitney t-test p-value of 0.0125 shows that the induced vasodilation effects are partly characterized by the release of nitric oxide from the vascular endothelial cells. Values are expressed as the mean ± SEM of six experiments (n = 6 aortas).

Figure 5 shows the effect of NG-nitro-L-arginine methyl-ester (L-NAME) on the vasodilatory effects of the SAPE plant extract.

## 4 Discussion and conclusion

The present study investigated the vasodilatory effects of crude polyphenolic compounds that were extracted and quantified as 952.00 mg GAE/100 g of *S. araliacea* root powdered material. Previous studies have demonstrated an association of stronger vasodilation with plant extracts of higher polyphenolic compounds (de Carvalho Emanuella et al., 2019; Jean-Marie et al., 2010; Mamadou et al., 2009). The value of 952 mg GAE/100 g derived from the standard gallic acid calibration curve in Figure 1 turned out to be the optimum yield from among the different extracting solvents used in the current study. Table 1 shows that 20% of methanol yielded the highest total polyphenol content of 952.00 mg GAE/100 g from the dry root powdered material of *S. araliacea* plant, and the least content was obtained in the chloroform extract, which was estimated as 97.78 mg GAE/100 g. This meant that chloroform yielded more non-polyphenolic compounds (non-polar solutes) in the extract, and thus, chloroform was then applied for partitioning of the methanolic extract to optimize the polyphenols and get rid of unwanted non-polar compounds. Therefore, methanol extract was used for subsequent investigations and characterization of the vasodilatory effects, and within the scope of this study, we investigated, among several other possibilities of mechanisms of action, the involvement of NO release for relaxation effects.

Figures 3, 4a,b have demonstrated that the methanolic extract of *S. araliacea* root polyphenols induces endothelium-dependent and endothelium-independent vasodilation on isolated rat aortas. The pattern is similar to that of the standard acetylcholine and sodium nitroprusside, as shown in Figures 2a–c.

The methanolic extract of *S. araliacea* was a crude sample comprising a cocktail of different phenolic compounds with different structures that could bind to diverse receptor sites in order to induce the vasodilation effect. This suggests a possible metabolite synergy for receptor activation by various polyphenolic metabolites present in the crude extract. Some of the metabolites may potentiate the effects of one to the other, while others may antagonize the desired effect.

While the concentration–response curves between endothelium-intact and denuded aortas might not be significantly different, the mere right-shift of the curve for endothelium-denuded aortas suggests a possible involvement of the endothelium-derived relaxing factors (EDRF), such as release of NO. Therefore, in the scope of this study, we endeavored to assess the possibility of vasodilation via the NO/cGMP pathway, as illustrated in Figure 5, showing the effect of the eNOS inhibitor, L-NAME, on the vasodilation effect of SAPE. The concentration–response curve in the presence of L-NAME (100 µM) shifted significantly to the right, showing a reduced pharmacological efficacy and the potency of SAPE, as evidenced by the increased IC_50_ values from 5.07 to 6.20 mg/mL. Mann–Whitney t-test p-value of less than 0.05 entails that the observed vasodilatory effects were partly induced by the release of NO from endothelial cells as a possible mechanism of action.

The relationship between the dose of a drug and its effect is a fundamental principle of pharmacology. In general, the effect of a drug increases as the dose increases, often in a non-linear fashion, reaching saturation levels at high concentrations of the drug. Similarly, drugs have a threshold below which they do not produce any effect, and a ceiling above which further increases in the dose do not lead to greater effects (Chung-Chuan et al., 2010). This dose–effect relationship is crucial for understanding how drugs work and for determining safe and effective dosages (Yagiela et al., 2010).

Our study findings agree with the reports of other studies (Fredi et al., 2019) regarding the polyphenolic composition of *Parastrephia quadrangularis* and its antihypertensive effects on rats, attributing the vasodilatory effects to a cocktail of polyphenols, in most medicinal plants, acting in synergy to stimulate generation of NO, which evokes the NO/cGMP pathway. Our results have also demonstrated and agreed to the report that polyphenol-rich natural edibles improve the hemodynamic flow in both physiological and pathological conditions (Dohadwala et al., 2011; Hampton et al., 2010; Clifto and n, 2004). However, the findings of this study conflict against one report (Fredalina et al., 2018) suggesting that the vasodilation by *Canarium odontophyllum* leaf extract does not involve the presence of EDRF because the vasodilatory effects by the dose–response curves were similar, though our findings do agree on the aspect of vasodilation being effected via endothelium-independent pathways. This is further espoused by similar studies (de Oliveira Lais et al., 2016) demonstrating significant vasorelaxation of denuded thoracic rat aortas with gallic acid.

The present study demonstrates that polyphenolic compounds extracted from *S. araliacea* induce significant vasorelaxation in isolated aortic rings, suggesting potential cardiovascular benefits. The vasorelaxant effects appear to be mediated, at least in part, through the NO pathway, as evidenced by the attenuation of relaxation in the presence of L-NAME, a non-selective NO synthase (NOS) inhibitor. NO is a critical vasodilator produced by endothelial cells via endothelial eNOS. Our findings indicate that the vasorelaxant effect of the *S. araliacea* polyphenol-rich extract was significantly reduced upon pretreatment with L-NAME, suggesting that NO plays a key role in the observed relaxation. This aligns with previous studies showing that many polyphenols, such as those from flavonoids and phenolic acids, enhance endothelial NO production, leading to vascular smooth muscle relaxation (Chen et al., 2024; Herbert, 2017).

The attenuation of vasorelaxation by L-NAME strongly implicates eNOS in the mechanism of action. Polyphenols are known to enhance eNOS activity through several mechanisms, including the phosphorylation of eNOS at Ser^1177^, activation of phosphoinositide 3-kinase (PI3)/protein kinase (PI3K/Akt) signaling, leading to eNOS phosphorylation and increased NO production, and prolongation of eNOS expression by preventing its degradation (Gabriele and Monica, 2023; Das et al., 2023; Kumiko et al., 2020).

While L-NAME significantly reduced vasorelaxation, the residual effect suggests NO-independent pathways, such as endothelium-derived hyperpolarizing factor (EDHF). Some polyphenols activate small- and intermediate-conductance Ca^2+^ and activated K^+^ channels, inducing hyperpolarization independently of NO (Teerapap et al., 2024). In addition, prostacyclin (PGI_2_) may contribute, and could be tested using indomethacin, along with the direct antioxidant effects of polyphenols by scavenging ROS, preserving NO bioavailability, and thereby enhancing vasodilation (Yeh Siiang et al., 2015).

The effects of *S. araliacea* polyphenols resemble those of well-characterized compounds such as resveratrol, which enhances eNOS activity by activating SIRT1; epicatechin, which increases NO via PI3K/Akt and Ca^2+^ -dependent eNOS activation; and quercetin, which modulates both NO and EDHF pathways (Jingbo et al., 2015; Ramirez-Sanchez et al., 2010; Ramirez-Sanchez et al., 2011; Khoo et al., 2010).

Given the critical role of endothelial dysfunction in hypertension and atherosclerosis, *S. araliacea* polyphenols could be promising agents for improving endothelial function in metabolic disorders (e.g., diabetes, hypertension, and atherosclerosis).

Future studies are recommended to identify the specific bioactive polyphenols, test *in vivo* acute and chronic administration in hypertensive models, and assess synergism with currently existing therapies. The limitations of the study include the use of aortic rings lacking neuro-hormonal influences, uncharacterized active compounds, and potential species differences between human and rat vascular responses.

In conclusion, the major finding of the present study is that the polyphenol crude extract of *S. araliacea* roots has the potency to induce both endothelium-dependent and independent vasodilation in thoracic rat aortas. We can safely report that SAPE has the properties to act as an antihypertensive agent through vascular myogenic tone reduction and is able to ameliorate the effects of endothelial dysfunction.

The vasorelaxant effects of *S. araliacea* polyphenols are primarily NO-dependent, mediated via eNOS activation and downstream cGMP-PKG signaling, as confirmed by L-NAME inhibition. However, NO-independent mechanisms (e.g., EDHF and antioxidant effects) may also contribute. These findings highlight the potential of *S. araliacea* polyphenolic extract as a natural vasodilator and warrant further mechanistic and translational studies.

Our study further reports that one possible avenue by which the vasodilatory effects are induced is by increasing the bioavailability of NO via the stimulation of eNO synthase activity on endothelium-intact aortas. This study further confirms the traditional use of the crude extract as a means to alleviate high blood pressure in human subjects; hence, the significance of this research work is the finding that plant natural products with antihypertensive properties are available and need extensive research, leading to potentially new drug leads. The extract was essentially non-toxic as no deaths were recorded.

## Data Availability

The datasets presented in this study can be found in online repositories. The names of the repository/repositories and accession number(s) can be found in the article/supplementary material.

## References

[B1] Aamer S. Van Veldhuijzen Z. J. J. C. S. Metsios G. S. Carroll D. Kitas George D. (2010). The endothelium and its role in regulating vascular tone. Open Cardiovasc. Med. J. 4, 302–312. 10.2174/1874192401004010302 21339899 PMC3040999

[B2] Agunu A. Abdurahman E. M. Andrew G. O. Muhammed Z. (2005). Diuretic activity of the stem-bark extracts of *Steganotaenia araliacea* hochst [Apiaceae]. J. Ethnopharmacol. 96 (3), 471–475. 10.1016/j.jep.2004.09.045 15619566

[B3] Agunu A. Ibrahim N. D. G. Onyiloyi G. A. Abdurahman E. M. (2003). Toxicity of stem-bark extract of *Steganotaenia araliacea* in rats. Niger. J. Nat. Prod. Med. 7, 65–67. 10.4314/njnpm.v7i1.11711

[B4] Akçay A. (2013). The calculation of LD_50_ using probit analysis. FASEB J. 27 (1_Suppl.), 1217.28. 10.1096/fasebj.27.1_supplement.1217.28

[B5] Andemariam S. W. (2010). Legislative regulation of traditional medicinal knowledge in Eritrea via-a-Vis Eritrea’s commitments under the convention on biological diversity: issues and alternatives. Law Env’t and Dev J. 6, 130.

[B6] Bernas G. C. Gonzales R. E. Solevilla R. C. Ysreal M. C. (2004). “Pharmacology - toxicology,” in Aguide to plant screening: phytochemical abd biological. Santo tomas. Editor Guevara B. (Philippines: University of Santo Tomas), 103–132.

[B7] Bi-Hui J. Ling-Bo Q. Chen S. Jun L. Hui-Ping W. Bruce I. C. (2009). Apigenin protects endothelium-dependent relaxation of rat aorta against oxidative stress. Eur. J. Pharmacol. 616 (1-3), 200–205. 10.1016/j.ejphar.2009.06.020 19549516

[B8] Bonaventura D. Lunardi C. N. Rodrigues G. J. Neto Mário A. Bendhack Lusiane M. (2008). A novel mechanism of vascular relaxation induced by sodium nitroprusside in the isolated rat aorta. Nitric Oxide 18 (4), 287–295. 10.1016/j.niox.2008.02.004 18307997

[B9] Bryda E. C. (2013). The mighty mouse: the impact of rodents on advances in biomedical research. Mo. Med. 110 (3), 207–211. Avaliable online at: https://pmc.ncbi.nlm.nih.gov/articles/PMC3987984/. 23829104 PMC3987984

[B10] Cavalcante J. L. Lima João A. C. Redheuil A. Al-Mallah M. H. (2011). Aortic stiffness: current understanding and future directions. J. Am. Coll. Cardiol. 57 (14), 1511–1522. 10.1016/j.jacc.2010.12.017 21453829

[B11] Chen G. Zhang L. Ann V. S. Xu W. (2024). Recent advances in activation of endothelial nitric oxide synthase by natural products: an effects and mechanisms review. Food Rev. Int. 40 (1), 260–275. 10.1080/87559129.2023.2166061

[B12] Chung-Chuan H. Bor-Yann C. Ding-Hung L. Chia-Chyi W. Yu-Ju T. (2010). Preliminary screening *via* dose–response analysis of the antibacterial activities of six Chinese medicinal plant extracts. J. Taiwan Inst. Chem. Eng. 41 (5), 579–584. 10.1016/j.jtice.2009.12.003

[B13] Clifton P. M. (2004). Effect of grape seed extract and quercetin on cardiovascular and endothelial parameters in high-risk subjects. J. Biomed. Biotechnol. 2004 (5), 272–278. 10.1155/S1110724304403088 15577189 PMC1082891

[B14] Council National Research, Earth Division on, Studies Life, Research Institute for Laboratory Animal (2011). Care committee for the update of the guide for the, animals use of laboratory. Guide for the care and use of laboratory animals. 8th edn. Washington, DC, United States National Academies Press.

[B15] Das M. Devi K. P. Tarun B. Prasad D. H. Devesh T. Adeleh S. (2023). Harnessing polyphenol power by targeting eNOS for vascular diseases. Crit. Rev. Food Sci. Nutr. 63 (14), 2093–2118. 10.1080/10408398.2021.1971153 34553653

[B16] de Carvalho Emanuella F. Nunes André F. Silva Náiguel C. B. da Silva Gomes João P. de Sousa Renato P. Silva Valdelânia G. (2019). *Terminalia fagifolia* Mart. & Zucc. elicits vasorelaxation of rat thoracic aorta through nitric oxide and K^+^ channels dependent mechanism. Biol. Open 8 (2), bio035238. 10.1242/bio.035238 30683674 PMC6398462

[B17] de Oliveira Lais M. de Oliveira Thiago S. da Costa Rafael M. de Souza G. E. Costa E. A. de Cassia Aleixo Tostes P. (2016). The vasorelaxant effect of gallic acid involves endothelium-dependent and-independent mechanisms. Vasc. Pharmacol. 81, 69–74. 10.1016/j.vph.2015.10.010 26643780

[B18] Dohadwala M. M. Holbrook M. Hamburg N. M. Shenouda S. M. Chung W. B. Titas M. (2011). Effects of cranberry juice consumption on vascular function in patients with coronary artery disease. Am. J. Clin. Nutr. 93 (5), 934–940. 10.3945/ajcn.110.004242 21411615 PMC3076649

[B19] Fastone G. Charity S. Penias T. Jr Musawa M. Theresa C. Longa K. (2021). May measurement month 2019: an analysis of blood pressure screening results from Zambia. Eur. Heart J. Suppl. 23 (Suppl. B), B158–B160. 10.1093/eurheartj/suab043 34054374 PMC8141953

[B20] Fredalina B. D. Sa’adah A. R. N. Ali Shafreena S. Satirah Z. (2018). The vasorelaxant effect of *Canarium odontophyllum* Miq. (Dabai) extract in rat thoracic aorta. Egypt. J. Basic Appl. Sci. 5 (1), 75–79. 10.1016/j.ejbas.2017.11.004

[B21] Fredi C. Javier P. Nwokocha Chukwuemeka R. Jorge B. Simirgiotis Mario J. Ignacio N. (2019). Polyphenolic composition and hypotensive effects of *Parastrephia quadrangularis* (Meyen) cabrera in rat. Antioxidants 8 (12), 591. 10.3390/antiox8120591 31783548 PMC6943605

[B22] Gabriele S. Monica D. (2023). Role of dietary polyphenols in the activity and expression of nitric oxide synthases: a review. Antioxidants 12 (1), 147. 10.3390/antiox12010147 36671009 PMC9854440

[B23] Gao Y. (2022). Biology of vascular smooth muscle: vasoconstriction and dilatation. Singapore: Springer Nature Singapore.

[B24] Goma F. M. Ezeala C. Nyirenda J. Chuba D. Prashar L. Simfukwe N. (2017). Extraction and demonstration of uterotonic activity from the root of *Steganotaenia araliacea* hochst. Med. J. Zamb. 44 (3), 125–132. 10.55320/mjz.44.3.80

[B61] Goma F. Syatalimi C. Tembo Jr P. Mukupa M. Chikopela T. Kaluba L. (2021). May Measurement Month 2019: an analysis of blood pressure screening results from Zambia. European Heart Journal Supplements, 23 (Supplement_B), B158–B160. 10.55320/mjz.44.3.80 34054374 PMC8141953

[B25] Hajj Magalie E. Doreen Chilolo S. Bellington V. Lone H. (2020). Herbal medicine use among pregnant women attending antenatal clinics in Lusaka Province, Zambia: a cross-sectional, multicentre study. Complementary Ther. Clin. Pract. 40, 101218. 10.1016/j.ctcp.2020.101218 32891293

[B26] Hampton S. M. Isherwood C. Kirkpatrick V. J. E. Lynne-Smith A. C. Griffin B. A. (2010). The influence of alcohol consumed with a meal on endothelial function in healthy individuals. J. Hum. Nutr. Dietetics 23 (2), 120–125. 10.1111/j.1365-277X.2009.01021.x 20113387

[B27] Herbert K. (2017). Beneficial vascular responses to proanthocyanidins: critical assessment of plant-based test materials and insight into the signaling pathways. Recent Adv. Polyphenol Res., 226–258. 10.1002/9781118883303.ch11

[B28] Hodge Harold C. Sterner James H. (1949). Tabulation of toxicity classes. Am. Industrial Hyg. Assoc. Q. 10 (4), 93–96. 10.1080/00968204909344159 24536943

[B31] Jean-Marie T. Cyril A. Lise B. Saliou N. ’G. Philippe C. Noureddine I.-K. (2010). Procyanidin-rich fractions from *Parkia biglobosa* (Mimosaceae) leaves cause redox-sensitive endothelium-dependent relaxation involving NO and EDHF in porcine coronary artery. J. Ethnopharmacol. 132 (1), 246–250. 10.1016/j.jep.2010.08.031 20727401

[B32] Jingbo L. Xie C. Zhuang J. Hali L. Yao Y. Shao C. (2015). Resveratrol attenuates inflammation in the rat heart subjected to ischemia-reperfusion: role of the TLR4/NF-κB signaling pathway. Mol. Med. Rep. 11 (2), 1120–1126. 10.3892/mmr.2014.2955 25405531

[B33] Kathy Lynn R. Matthew G. F. Amelia B. J. Ann O. R. Scott F. M. Douglas S. (2018). Hypertension prevalence and risk factors in rural and urban Zambian adults in western province: a cross-sectional study. Pan Afr. Med. J. 30 (1), 97. 10.11604/pamj.2018.30.97.14717 30344881 PMC6191248

[B34] Khoo N. K. H. Roger W. C. Pozzo-Miller L. Zhou F. Chad C. Takafumi I. (2010). Dietary flavonoid quercetin stimulates vasorelaxation in aortic vessels. Free Radic. Biol. Med. 49 (3), 339–347. 10.1016/j.freeradbiomed.2010.04.022 20423726 PMC2900862

[B35] Kumiko T. Ikumi T. Kaneko N. Matsumoto T. Kobayashi T. (2020). Plant polyphenols morin and quercetin rescue nitric oxide production in diabetic mouse aorta through distinct pathways. Biomed. and Pharmacother. 129, 110463. 10.1016/j.biopha.2020.110463 32768953

[B36] Lassale C. Gaye B. Diop I. B. Mipinda J. B. Kramoh K. E. Kouam C. K. (2022). Use of traditional medicine and control of hypertension in 12 African countries. BMJ Glob. Health 7 (6), e008138. 10.1136/bmjgh-2021-008138 35654446 PMC9163537

[B37] Liu C. Huang Y. (2016). Chinese herbal medicine on cardiovascular diseases and the mechanisms of action. Front. Pharmacol. 7, 469. 10.3389/fphar.2016.00469 27990122 PMC5130975

[B38] Mamadou S. Saliou N. Kane Modou O. Alassane W. Doudou D. Bocar S. (2009). *In vitro* vasorelaxation mechanisms of bioactive compounds extracted from *Hibiscus sabdariffa* on rat thoracic aorta. Nutr. and metabolism 6, 1–12. 10.1186/1743-7075-6-45 PMC2777910 19883513

[B39] Mazuru G. (2019). Evaluation of anticancer activity of *Steganotaenia araliacea* (carrot tree) bark extract in cancer induced mammary glands of female sprague dawley rats. Int. J. Humanit. Arts, Med. Sci. 7 (1), 7–16.

[B40] Mona A. Tannaz J. Arrigo C. Vanessa B. Matteo P. Maciej B. (2020). The potential role of plant-derived natural products in improving arterial stiffness: a review of dietary intervention studies. Trends Food Sci. and Technol. 99, 426–440. 10.1016/j.tifs.2020.03.026

[B41] Mussie Sium D. Patrick G. K. Joseph M. K. Girmay N. B. (2014). GC-MS analysis of the essential oil and methanol extract of the seeds of *Steganotaenia araliacea* hochst. Am. J. Plant Sci. 5 (26), 3752. 10.4236/ajps.2014.526392

[B42] Nino J. Nishat A. Tripathi Y. C. (2016). Phytochemical screening and evaluation of polyphenols, flavonoids and antioxidant activity of *Prunus cerasoides* D. Don leaves. J. Pharm. Res. 10 (7), 502–508.

[B59] Nyirenda J. Chipuwa M. (2024). An ethnobotanical study of herbs and medicinal plants used in Western, Copperbelt, Central and Northern provinces of Zambia. Phytomedicine Plus, 4 (1), 100514.

[B60] Nyirenda J. Kadango Z. Funjika E. Chipabika G. (2024). Larvicidal, ovicidal and antifeedant activity of crude cashew nutshell liquid against fall armyworm, Spodoptera frugiperda (JE smith), (lepidoptera, Noctuidae). Crop Protection. 179, 106619.

[B43] Omolo J. J. Maharaj V. Dashnie N. Malebo H. M. Mtullu S. Lyaruu H. V. M. (2014). Flavonoids of *Steganotaenia araliacea* . Am. J. Res. Commun. 2 (8), 52–60.

[B44] Onwusonye J. C. Uwakwe A. A. Iwuanyanwu P. Iheagwam U. (2014). Oral acute toxicity (LD_50_) study of methanol extract of *Annona senegalensis* leaf in albino mice. Sky J. Biochem. Res. 3 (5), 46–48.

[B45] Pharaoh H. Mathew G. F. Kennedy C. Newton S. Lukubi L. Chitembusha M. K. (2021). Effects of *Steganotaenia araliacae* root extract on contractile function of isolated rat ileum. J. Prev. Rehabilitative Med. 3 (2), 32–41. 10.21617/jprm2021.328issn

[B46] Ramirez-Sanchez I. Lisandro M. Guillermo C. Francisco V. (2010). (−)-Epicatechin activation of endothelial cell endothelial nitric oxide synthase, nitric oxide, and related signaling pathways. Hypertension 55 (6), 1398–1405. 10.1161/HYPERTENSIONAHA.109.147892 20404222 PMC2874202

[B47] Ramirez-Sanchez I. Lisandro M. Guillermo C. Francisco V. (2011). (−)-Epicatechin induces calcium and translocation independent eNOS activation in arterial endothelial cells. Am. J. Physiology-Cell Physiology 300 (4), C880–C887. 10.1152/ajpcell.00406.2010 PMC3074631 21209365

[B48] Rica C. I. An W. Kenn F. Baldé A. M. Sandra A. Filip L. (2015). Phytochemical characterisation of a cytotoxic stem bark extract of *Steganotaenia araliacea* and identification of a protoflavanone by LC–SPE–NMR. Phytochem. Lett. 12, 119–124. 10.1016/j.phytol.2015.03.008

[B49] Sánchez-Rangel J. C. Benavides J. Heredia J. B. Cisneros-Zevallos L. Jacobo-Velázquez D. A. (2013). The Folin–Ciocalteu assay revisited: improvement of its specificity for total phenolic content determination. Anal. Methods 5 (21), 5990–5999. 10.1039/c3ay41125g

[B50] Singleton Vernon L. Rudolf O. Lamuela-Raventós Rosa M. (1999). [14] Analysis of total phenols and other oxidation substrates and antioxidants by means of folin-ciocalteu reagent. Methods Enzym. 299, 152–178. 10.1016/s0076-6879(99)99017-1

[B51] Teerapap P. Kornkanok I. Krongkarn C. Prapapan T. Nungruthai S. Maude T.-N. (2024). Vasorelaxant and hypotensive effects of an ethanolic extract of Nymphaea pubescens and its main compound quercetin 3-methyl ether 3′-O-β-xylopyranoside. Front. Pharmacol. 15, 1379752. 10.3389/fphar.2024.1379752 38576494 PMC10991828

[B52] Touyz R. M. Alves-Lopes R. Rios F. J. Camargo Livia L. Anagnostopoulou A. Arner A. (2018). Vascular smooth muscle contraction in hypertension. Cardiovasc. Res. 114 (4), 529–539. 10.1093/cvr/cvy023 29394331 PMC5852517

[B53] Vaithiyanathan V. Mirunalini S. (2015). Assessment of antioxidant potential and acute toxicity studies of whole plant extract of *Pergularia daemia* (Forsk). Toxicol. Int. 22 (1), 54–60. 10.4103/0971-6580.172257 26862261 PMC4721177

[B54] Vivian Caetano B. Teixeira B. M. Rodrigues D. Caroline Sefrin S. Monica G. M. Teixeira G. H. (2014). Polyphenol extraction optimisation from ceylon gooseberry (*Dovyalis hebecarpa*) pulp. Food Chem. 164, 347–354. 10.1016/j.foodchem.2014.05.031 24996344

[B55] Welfare National Institutes of Health (2002). Office of laboratory animal, association applied research ethics national. Institutional animal care and use committee guidebook: office of laboratory animal welfare.

[B56] Yagiela J. A. Dowd Frank J. Bart J. Angelo M. Neidle E. A. (2010). Pharmacology and therapeutics for dentistry-E-Book: pharmacology and therapeutics for dentistry-E-Book. Elsevier Health Sciences.

[B57] Yeh Siiang L. Chih L. W. Dharmani M. Rais M. M. (2015). Boldine ameliorates vascular oxidative stress and endothelial dysfunction: therapeutic implication for hypertension and diabetes. J. Cardiovasc. Pharmacol. 65 (6), 522–531. 10.1097/FJC.0000000000000185 25469805 PMC4461386

[B58] Yong-He Z. Yang-Su P. Tack-Joong K. Lian-Hua F. Hee-Yul A. JinTae H. (2000). Endothelium-dependent vasorelaxant and antiproliferative effects of apigenin. General Pharmacol. Vasc. Syst. 35 (6), 341–347. 10.1016/s0306-3623(02)00113-1 11922965

